# Effects of transforming growth factor β-1 infected human bone marrow mesenchymal stem cells on high- and low-metastatic potential hepatocellular carcinoma

**DOI:** 10.1186/s40001-015-0144-2

**Published:** 2015-05-24

**Authors:** Tianran Li, Shaohong Zhao, Bin Song, Zhengmao Wei, Guangming Lu, Jun Zhou, Tianlong Huo

**Affiliations:** Department of Radiology, The 95th Hospital of PLA, 485 Dongyan Road, Putian, Fujian province 351100 People’s Republic of China; Department of Radiology, The 304th Hospital of PLA, 51 Fucheng Road, Beijing, Haidian District 100048 People’s Republic of China; Department of Radiology, Peking University People’s Hospital, 11 South street of Xizhimen, Beijing, Xicheng District 100048 People’s Republic of China; Department of Radiology, The Nanjing General Hospital of PLA, Nanjing, Jiangsu province 21000 People’s Republic of China

**Keywords:** hMSC, Hepatoma cells, TGFβ-1, Genetically modified, OPN, PDCD4

## Abstract

**Background:**

This study investigates the effects of human bone marrow-derived mesenchymal stem cell (hMSC) on migration and proliferation ability of hepatocellular carcinoma (HCC) with high- and low-metastatic potential.

**Methods:**

The hMSC and transforming growth factor-β1 (TGFβ-1) gene infected hMSC were co-cultured with hepatoma cells. The ability of cells migration was assessed by Transwell assay. The ability of cells proliferation was detected using CCK-8 assay. The mice were engrafted with hMSC and TGFβ-1 gene infected hMSC, respectively, after hepatoma cells inoculation 15 days, twice a week for 6 weeks successively. The tumor inhibition rate was calculated. TGFβ-1, osteopontin (OPN), and programmed cell death protein 4 (PDCD4) genes expression of hepatoma cells were detected by quantitative real-time polymerase chain reaction (qPCR) before and after co-cultured experiments.

**Results:**

TGFβ-1 infected hMSC or hMSC co-culture with hepatoma cells groups can significantly promote hepatoma cells proliferation (*P* < 0.05). The migration numbers of hepatoma cells with TGFβ-1 infected hMSC co-culture groups were significantly reduced compared with the other two groups (*P* < 0.05). The tumors weight inhibition rates of MHCC97-H and MHCC97-L animal models were the highest in the third week by hMSC engraftment. But the highest tumor inhibition rate of MHCC97-H animal models was observed in the fourth week and MHCC97-L animal models in the fifth week after TGFβ-1 infected hMSC engraftment. OPN gene relative quantitative expression of hepatoma cells was significantly down-regulated after co-cultured with hMSC and TGFβ-1 gene infected hMSC groups (*P* < 0.05). TGFβ-1 gene relative quantitative expression of MHCC97-H and MHCC97-L cells was significantly up-regulated after co-cultured with TGFβ-1 gene infected hMSC groups (*P* < 0.05). PDCD4 expression had no statistical differences among groups.

**Conclusions:**

hMSC and TGFβ-1 gene infected hMSC can promote hepatoma cells proliferation and inhibit hepatoma cells migration. hMSC and TGFβ-1 gene infected hMSC exhibit anti-tumor activity in a time-dependent manner. TGFβ-1 cytokine may be the main factor in HCC proliferation. OPN makes a significant contribution to the changes of hepatoma cells metastasis.

## Background

Hepatocellular carcinoma (HCC) is one of the most common cancers with poor prognosis and high recurrence rate, and metastatic recurrence is the major obstacle to improve the prognosis of HCC patients. Human bone marrow-derived mesenchymal stem cell (hMSC) is a type of adult stem cells with multilineage differentiation potential. Previous studies have reported that hMSCs could inhibit the growth of hepatocellular carcinoma tissue and repair the impaired liver tissue. Hence, hMSCs become a hot-spot for the treatment of hepatocellular carcinoma [1, 2]. However, there is also the opposite view. Researchers believed that hMSC can promote the growth and metastasis of hepatocellular carcinoma cells [3]. Furthermore, the metastasis of hepatocellular carcinoma is a key factor for its prognosis, and it is important to extend the life-span of HCC patients by observing the effect of hMSCs on HCC metastatical ability. Enhancing hMSC resistance ability to HCC tissue and metastatic potentials by genic engineering technology has been regarded as a feasible way. It has been reported that transforming growth factor beta (TGFβ) is a multifunctional cytokine family, which mainly plays roles in regulating cell proliferation, differentiation, and embryo development, promoting the formation of extracellular matrix and inhibiting immune response. Among the members in this family, serum TGFβ-1 is a principal isoform in humans, and it is closely associated with the occurrence and development of tumor. TGFβ-1 plays dual roles in inhibiting and promoting tumor growth. At the early stage of tumorogenesis, TGFβ-1 inhibited normal cell growth and tumorogenesis by suppressing G1/S phase transition [4, 5]. However, the tumor would not be sensitive to TGFβ-1 mediated growth inhibition with the development of tumor. As a tumor growth stimulating factor, TGFβ-1 plays an important role in tumor growth, invasion, and metastasis. Therefore, in order to investigate intervention effects of hMSC to hepatocellular carcinoma tissue, in this study, TGFβ-1 was infected into human bone marrow-derived mesenchymal stem cells using a transgenic technology based on the biological characteristics of TGFβ-1. Our study mainly focuses on the effects of TGFβ-1 infected hMSC on HCC cells with high- and low-metastatic potentials, and the roles of hMSC in HCC progression in vitro are determined. Our data provide a preliminary exploration of the treatment against hepatocellular carcinoma metastasis using hMSC by genic engineering technology.

## Methods

### Reagents

Fetal calf serum (FCS) was purchased from PAN (Aidenbach, Germany), and trypsin tenfold was supplied by PAA (Pasching, Austria). Dulbecco’s modified eagle’s medium (DMEM) was supplied by Dulbecco’s and α-minimum essential medium (MEM) was supplied by ATCC.

### Cell lines

Human hepatocellular carcinoma cell lines with high-metastatic potential (MHCC97-H) and low-metastatic potential (MHCC97-L) were provided by the Liver Cancer Institute of Fudan University (Shanghai, China). hMSC were purchased from the Cyagen Biotech Co. Ltd. (Guangzhou, China).

### Animals

Thirty-six 6-week aged specific-pathogen-free (SPF) grade nude mice of the Balb/c strain (female to male ratio = 1), each weighing 14–17 g, were purchased from the Laboratory Animal Center of National Institutes for Food and Drug Control (Beijing, China). Animals were kept within the animal care facility of the Peking University Health Science Center. The experiments conform to the Guide for the Care and Use of Laboratory Animals published by the US National Institutes of Health (NIH Publication No. 85–23, revised 1996). The housing and care and procedures in the study were performed in accordance with the guidelines and regulations composed by the Animal Care Committee of the University of the Peking University Health Science Center and approved by the Institutional Animal Care and Use Committee of the Peking University Health Science Center, China.

### Hepatoma cells and hMSCs culture

MHCC97-H and MHCC97-L cells were routinely cultured on 75-cm^2^ culture flasks in high-glucose DMEM supplemented with 10 % FCS, 1 % L-glutamine, and 1 % penicillin/streptomycin at 37 °C and 5 % CO_2_ in a humidified incubator. The medium was replaced at 50 % of cell confluence. Tumor cells were passaged once (80 % of cell confluence), cultured for a further 2 days, and then trypsinized for subsequent implantation studies. Tumor cells from the same passage were used for all the implantation experiments.

The hMSCs were washed once with α-MEM and seeded at a concentration of 1 × 10^6^ cells/cm^2^ per 100-mm cell culture dish (Corning, Corning, NY) in α-MEM media containing 10 % fetal calf serum, nonessential amino acids (Cellgro, Herndon, VA), and pyruvate (Invitrogen, Carlsbad, CA) and cultured at 37 °C in 5 % CO_2_. After 48 h, nonadherent cells were removed, fresh media was added, and the culture was maintained for 7 days. Before infusion, cells were washed twice with phosphate buffer solution (PBS) and harvested and using magnetic beads (Miltenyi Biotech, Auburn, CA). Cells were resuspended at a concentration of 5 × 10^6^cells/ml. Expanded cells that displayed morphological, immunophenotypical, and differentiation properties of mesenchymal stem cells were identified again. Flow cytometry were used to observe the cluster of differentiation (CD) of hMSC. Cy3 and hoechst33342 (Sigma) labeled hMSC to observe proliferation under fluorescence microscopes. Cy3 excitation wavelength is 550 nm, and emission wavelength is 565 nm. hoechst33342 excitation wavelength is 350 nm, and emission wavelength is 461 nm.

### TGFβ-1 gene infecting hMSC

First is the construction of retroviral vector. The pLV.EX3d.P/neo-EF1A > TGFβ-1 > IRES/eGFP shuttling plasmid was constructed using Gateway technology. Overlap extension quantitative real-time polymerase chain reaction (qPCR) was used to amplify attB1-Kozak-TGFβ-1-attB2.

Primer sequences were as follows:

attB1-K-TGFβ-1, 5′-GGGGACAAGTTTGTACAAAAA AGCAGGCTGCCACCATGCCGCCCTCCGGGCTG-3′; attB2-TGFβ-1, 5′-GGG GACCACTTTGTACAAGAAAGCTGGGTTCAGCTGCACTTGCAGGAGCG-3′. Subsequently, pDown-TGFβ-1 plasmid and pLV.EX3d.P/neo-EF1A > TGFβ-1 > IRES/eGFP plasmid were constructed. The positive clone was screened using colony PCR, and the positive plasmids were further sequenced.

Second, lentivirus was produced. Totally, 5 × 10^6^ well-growth 293FT cells were counted and seeded into a 10-cm culture dish overnight. The old medium was removed, and 5 ml of DMEM containing 10 % FCS was added. Plasmid pLV.EX3d.P/neo-EF1A > TGFβ-1 > IRES/eGFP was added into the culture medium. The medium was substituted 24 and 48 h after the infection, respectively. The viruses were collected and concentrated 72 h after the infection, and the viral titre was further measured. Moreover, the cells were stained with crystal violet after drug screening.

Third is the TGFβ-1 infecting of hMSC. One milliliter of fresh complete medium of hMSCs was added into each well. Totally, 30 μl of viruses was used to infect the cells in one well, and the other well was set as blank control. The cells were cultured after mixing. The complete medium containing viruses was removed after 16 h of infection, and the cells were washed with PBS twice. The cells were continuously cultured for 72 h, and the infection efficacy was observed using a fluorescence microscope. The cells were used to perform PCR and western blot when the infection rate met the requirements. And hMSCs were observed using immunofluorescence before and after the infection.

### Assessment of tumor cell proliferation

Cell counting kit (CCK-8) assay is used to evaluate the proliferation ability of hepatoma cells. Dimethyl sulfoxide (DMSO) in 3-(4, 5)-dimethylthiahiazo(-z-y1)-3,5-di-phenytetrazoliumromide (MTT) assay would affect the culture membrane; therefore, we detected cell proliferation using CCK-8 assay. Totally, 2 × 10^3^ tumor cells (75 μl) were seeded into each upper chamber in a 96-well plate after the hMSC cells were adhered to the lower chamber. After 48 h of co-culture, the medium in each well was supplied to 80 μl in total, and 8 μl of CCK-8 was added into each upper chamber well. The mixture was incubated at 37 °C for 4 h. After the incubation, the optical density (OD) at 490 nm was detected using an ELISA reader. The experiment is repeated for six times.

### Assessment of tumor cell migration

The basement membrane (BM) is a specialized form of extracellular matrix (ECM), which is a major barrier to tumor cells during metastasis. Transwell assay is used to evaluate that the hepatoma cells break through the basement membrane (or migration) ability. ECM gel (Sigma, Swiss) was precooled at 4 °C for 2 h, and 50 μl of ECM gel was added into the upper chamber. The chamber was incubated at 37 °C in a humidified atmosphere of 5 % C0_2_ until the gel was solidified. The tumor cells were cultured with serum-free medium for 24 h and were counted after digestion. Moreover, these cells were resuspended using medium supplemented with 0.1 % FCS, and the cell concentration was adjusted to 1 × 10^6^ cells/ml. Totally, 10,000 human mesenchymal stem cells were seeded in the lower chamber using 500 μl of medium supplemented with 20 % FCS, and 100 μl of hepatoma cells suspension at a concentration of 1 × 10^6^ cells/ml was added into the upper chamber. ECM gel chamber was incubated at 37 °C in a humidified atmosphere of 5 % CO_2_ for 36 h. The cells on the upper surface of chamber were removed. Next, 500 μl of MTT (0.5 mg/ml) was added into each well of a 24-well plate. The chambers were immersed in medium at 37 °C for 4 h. Then, 500 μl of DMSO was added into each well, and the chambers were immersed in DMSO and oscillated for 10 min. Finally, formazan crystal was completely dissolved. The chambers were got out, and 100 μl DMSO was transferred into each well of a 96-well plate. The tumor cells on ECM gel and in upper chamber were removed using cotton swabs. The tumor cells were stained using 0.1 % crystal violet. The tumor cells were counted using a common microscope, and the chamber was everted to clearly observe the tumor cells in the lower chamber. The experiment is repeated for six times.

### Animal procedures

Mice were randomly assigned into one of two groups (*n* = 15 animals per group) in MHCC97-H and MHCC97-L group, and all mice were weighed and numbered. The mice in the experimental group were engrafted with hMSCs (5 × 10^5^ cells per mouse) via the tail vein 15 days after inoculation of tumor cells, twice a week for 6 weeks successively, while the animals in the control group were injected with hMSC culture medium (0.2 ml per mouse) via the tail vein at the same time. The subcutaneous tumor size was measured using an electronic digital caliper once every 4 days after hMSC engraftment. After 2, 3, 4, 5, and 6 weeks of tumor cell inoculation, the mice were killed and the tumors were collected in their entirety. The tumor weight and body weight of mice were measured.

The tumor inhibition rate was calculated using the following formula:

$$ \mathrm{Tumor}\kern0.5em \mathrm{inhibition}\kern0.5em \mathrm{rate}\left(\%\right)=\left[1-\frac{E\left(\mathrm{g}\right)}{C\left(\mathrm{g}\right)}\right]\times \mathsf{100}\% $$

*E* (g), mean tumor weight in the experiment group; *C* (g): mean tumor weight in the control group.

### Relative quantification using quantitative RT-PCR analysis

The primer sequences were obtained from GenBank database and synthesized by Cyagen Biotech Co. Ltd. (Guangzhou, China). The primer sequences of these genes were as follows (see Table 1).

**Table 1:** Invasion and proliferation-related gene relative quantitative expression of hepatoma cells

	OPN		TGFβ-1		PDCD4	
	MHCC97-H (%)	MHCC97-L (%)	MHCC97-H (%)	MHCC97-L (%)	MHCC97-H (%)	MHCC97-L (%)
Medium	100	100	100	100	100	100
hMSC	75.23	83.14	157.54	129.04	69.61	64.33
TGFβ-1/hMSC	51.03	49.11	206.52	176.52	233.31	125.45

β-Actin and GAPDH was used for normalization. Totally, 3 × 10^4^ hMSC cells that were seeded into the lower chamber of a 12-well plate after MHCC97-H cells were adhered to the upper chamber. After 48 h of co-culture, cells of the upper chamber were harvested for PCR detection. Total RNA was extracted, and cDNA was obtained using RevertAid ™ M-MLV RT system and Oligo (dT). The specific cDNA was amplified using PCR, and the profile was as follows: 95 °C for 5 min, followed by 30 cycles of 94 °C for 30 s, 58 °C for 30 s, and 72 °C for 30 s, with a final extension at 72 °C for 10 min. The PCR products were semi-quantitatively analyzed using gel electrophoresis.

### Statistics

Data were analyzed using Statistical Product and Service Solutions software (SPSS, version 16.0). Results were expressed as mean ± standard error of the mean (SEM). Differences between two groups were evaluated with the unpaired Student’s *t* test. Analysis of variance (ANOVA) was used to determine statistical differences. A *p* value < 0.05 was considered significant.

## Results

### Mesenchymal stem cells identification results

**Fig. 1:**
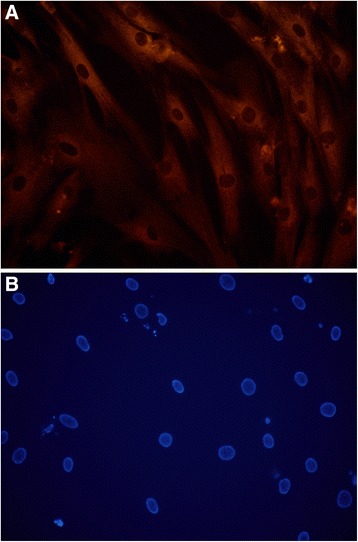
hMSC type I collagen Cy3 immunofluorescence staining and nucleus hoechst33342 staining. **a** *Red* represents the Cy3 immunofluorescence staining positive cells skeleton. hMSCs are spindle-shaped, the size are uniform, and the polarity is arranged. **b** *Blue* represents the hMSC nucleus. The nuclei are round, oval shape. Scale bars = 50 μm for (**a**–**b**)

hMSCs growth curve was drawn from the first day they recovered. The morphology, growth and proliferation of the cells were observed during continuous culture in vitro, and doubling time of cells was calculated. According to growth curve of experiment data to calculate the cells, doubling time is 26 h. After pass-generation tests for five times, the cells still has the vigor. At a ratio of 1:2 cells inoculated, cells can be covered within 72 h. In morphological observation under microscope and fluorescence microscopes (Fig. 1a, b), hMSCs are spindle-shaped, the size are uniform, and the polarity is arranged. Distribution of collagen in the cytoplasm and the nucleus shape are normal. The expressions of surface antigens CD29, CD44, and CD105 on these cells were detected by Flow Cytometry, and cells did not express CD45 and CD14 surface antigens. So, the experimental hMSC conformed to standards made by The International Society for Cellular Therapy position statement (2006) [6].

### TGFβ-1 gene infection of hMSC

**Fig. 2:**
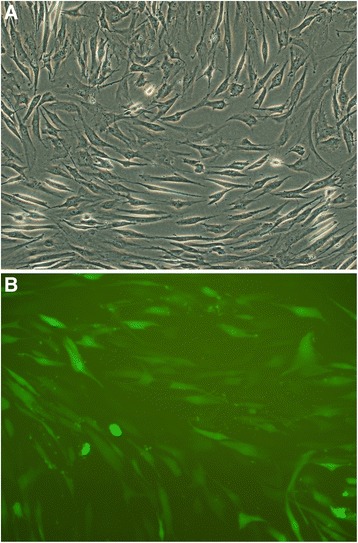
hMSC imaging of hMSC infected TGFβ-1 gene with reporter gene GFP observed under fluorescence microscope and inverted microscope. **a** hMSCs which have been penetrated by the green fluorescent protein (GFP) begin to glow with *bright green*. Original magnification. **b** Cells arranged regularly. The shape of gene-transduced hMSC was essentially the same as no-genetically modified cells. Original magnification. Scale bars = 100 μm for (**a**), 50 μm for (**b**)

First, morphological change of infected hMSCs was observed. Green fluorescent protein (GFP) was used as a reporter gene, and the target gene infected hMSC was observed under the fluorescence microscope compared with without gene infected hMSC (Fig. 2a,b).

**Fig. 3:**
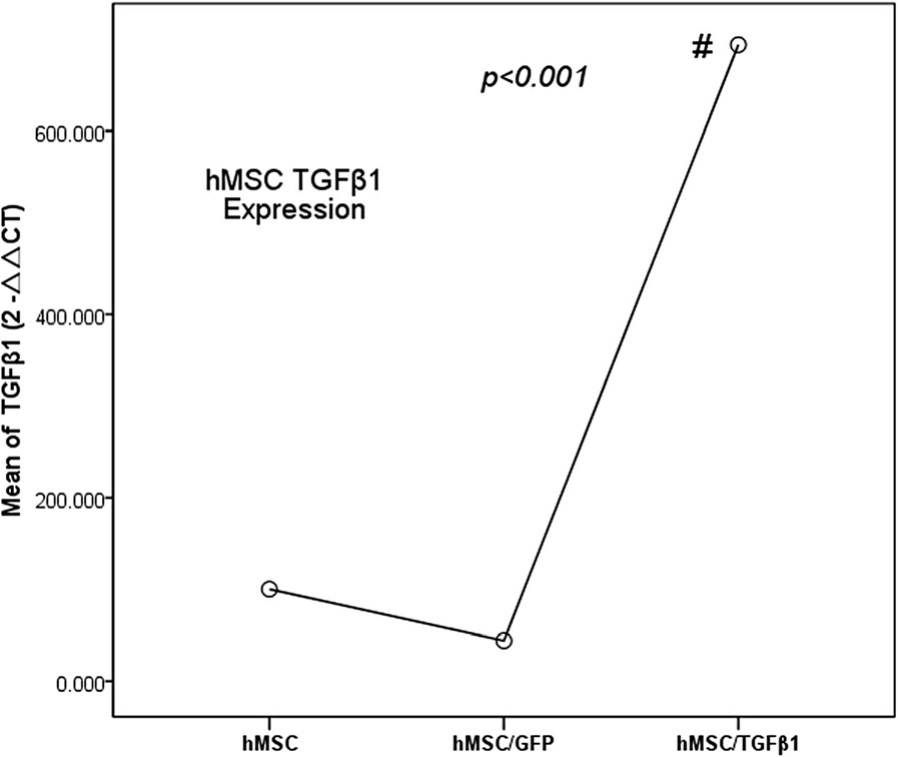
TGFβ-1 expression changes were detected using qPCR before and after hMSC infected target gene. The TGFβ-1 expression of hMSC infected TGFβ-1 gene group was higher than that of control groups (#*p* < 0.001). But the difference between hMSC and hMSC infected GFP group had not statistical significance (*p* > 0.05)

Second, TGFβ-1 gene expression changes of hMSC were detected by qPCR before and after the infection target gene. hMSC and hMSC infected GFP are the control groups; hMSC infected TGFβ-1 is the experimental group (Fig. 3).

Figure 3 showed that the TGFβ-1 expression of hMSC infected TGFβ-1 gene group was higher than that of control groups ((#*p* < 0.001), and TGFβ-1 expression of experimental group was about seven times as that of blank control group. But the difference between hMSC and hMSC infected GFP group had no statistical significance (*p* > 0.05). It has been indicated that the hMSC infected TGFβ-1 gene was successful and can proceed to the next step.

### Assessment of hMSC on hepatoma cells proliferation

**Fig. 4:**
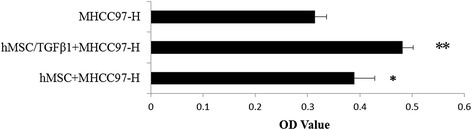
TGFβ-1 infected hMSC co-culture with MHCC97-H cells proliferation test. OD value of TGFβ-1 infected hMSC co-culture with MHCC97-H cell group was significantly higher than that of other groups (**p < 0.05). OD value of hMSC co-culture with MHCC97-H cell group was higher than that of control group (*p < 0.05)

**Fig. 5:**
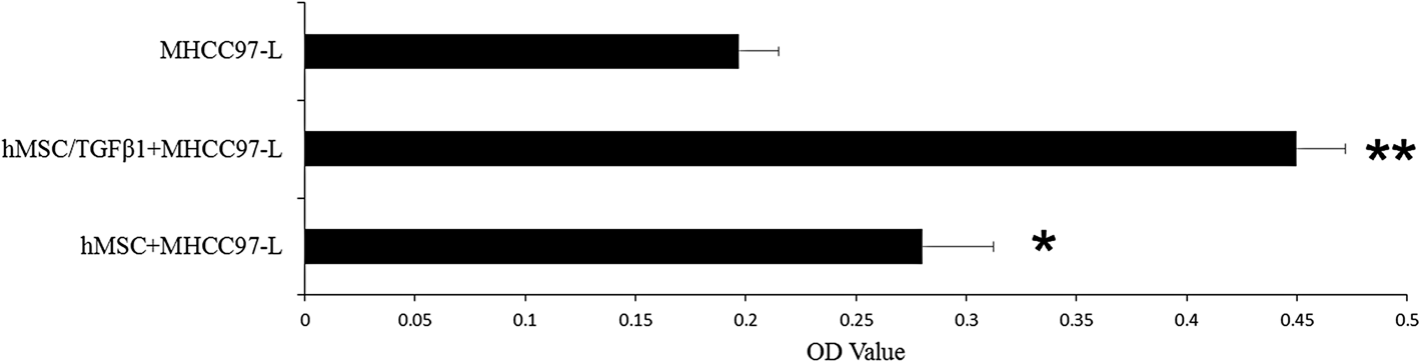
TGFβ-1 infected hMSC co-culture with MHCC97-L cells proliferation test. OD value of TGFβ-1 infected hMSC co-culture with MHCC97-L cell group was significantly higher than that of other groups (**p < 0.05). OD value of hMSC co-culture with MHCC97-L cell group was higher than that of control group (*p < 0.05)

The proliferation ability effects of hMSC and TGFβ-1 infected hMSC on hepatoma cells (MHCC97-H and MHCC97-L) were detected using CCK-8 (Cell Counting Kit-8) assay. hMSC and TGFβ-1 infected hMSC co-culture with hepatoma cells groups served as experimental groups. MHCC97-H and MHCC97-L groups served as control groups (Figs. 4 and 5).

As shown in Figs. 4 and 5, hMSC can promote MHCC97-H and MHCC97-L cells proliferation, especially TGFβ-1 gene infected hMSC.

### Assessment of hMSC on hepatoma cells migration

**Fig. 6:**
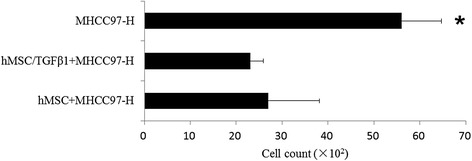
MHCC97-H cells migration counting of before and after TGFβ-1 infected hMSC co-culture with MHCC97-H. The migration numbers of hepatoma cells with TGFβ-1 infected hMSC co-culture groups were significantly reduced compared with those of the control group (*p < 0.05)

**Fig. 7:**
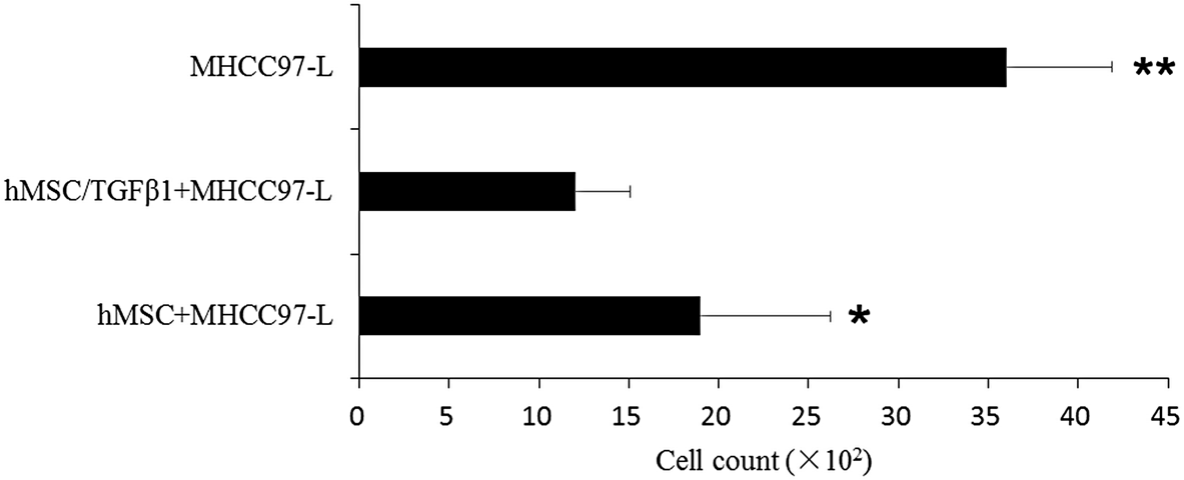
Cells migration counting of before and after TGFβ-1 infected hMSCs co-culture with MHCC97-L. The migration numbers of hepatoma cells with TGFβ-1 infected hMSC co-culture groups were significantly reduced compared with the other two groups (**p < 0.05), and TGFβ-1 infected hMSC co-culture group was significantly reduced compared with hMSC co-culture group (*p < 0.05)

The two hepatoma cells were co-cultured with hMSC and TGFβ-1 infected hMSC, respectively, and hepatoma cells with culture medium acted as the control groups. The breaking through basement membrane migratory hepatoma cells were stained using 0.1 % crystal violet. The migration of hepatoma cells was evaluated by means of a quantitative counting procedure (CCK-8) (Figs. 6 and 7).

The migration numbers of MHCC97-H cells with TGFβ-1 infected hMSC co-culture groups were significantly reduced compared with the control group (*p < 0.05).

The migration numbers of MHCC97-L cells with TGFβ-1 infected hMSC co-culture groups were significantly reduced compared with the other two groups (**p < 0.01), and TGFβ-1 infected hMSC co-culture with MHCC97-L cells group was significantly reduced compared with hMSC co-culture group (*p < 0.05).

### Animal model experiment results

**Fig. 8:**
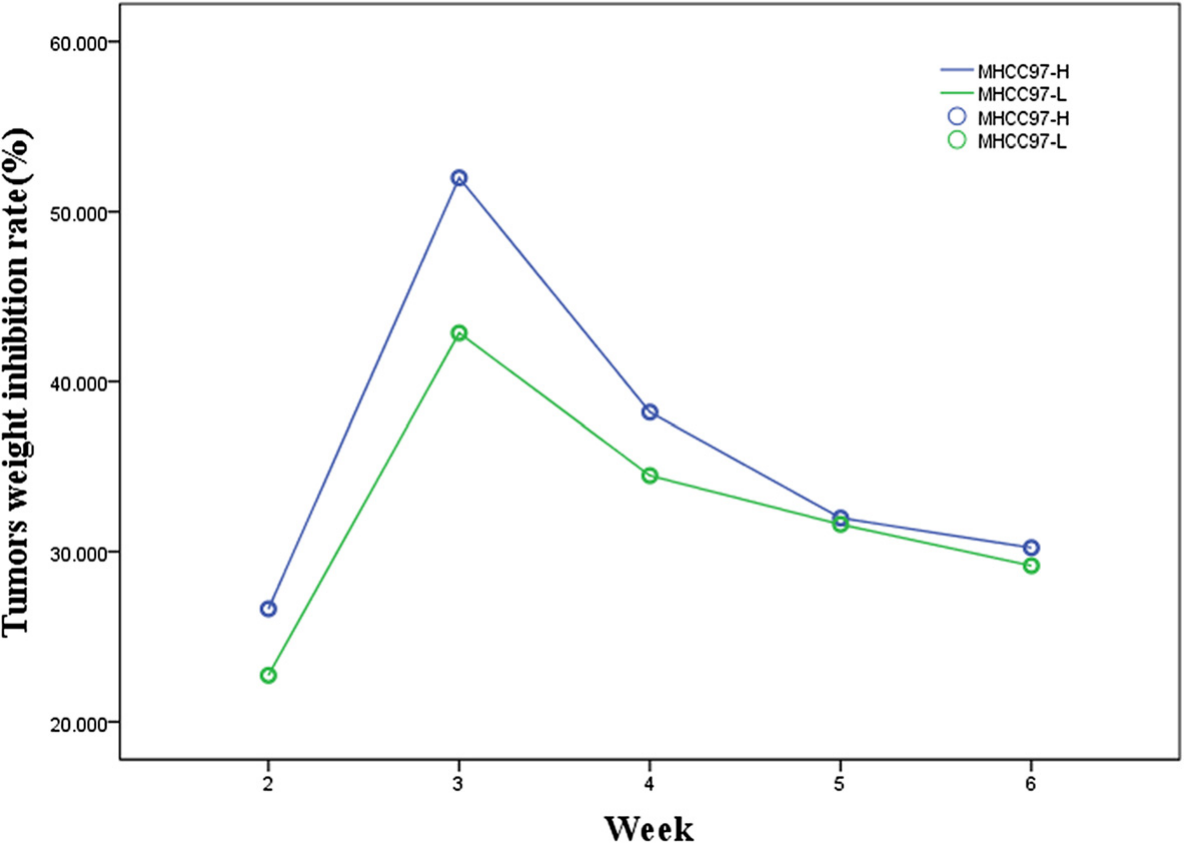
The changes of HCC animal model tumors weight inhibition rate following engraftment of hMSC over time. The tumors weight inhibition rates of MHCC97-H (*blue*) and MHCC97-L (*green*) animal models were the highest in the third week by hMSC engraftment

Firstly, effects of hMSC on transplanted tumors in nude mice produced by inoculation of MHCC97-H and MHCC97-L cells were investigated. The experiment lasted for 6 weeks. According to the tumor tissue specimens’ weight in vitro before and after hMSC intervention, the tumor weight inhibition rate was calculated (Fig. 8).

It was observed that the highest tumor inhibition rate was observed 3 weeks after hMSC engraftment, and the tumor inhibition rate gradually reduced with the prolongation of time.

**Fig. 9:**
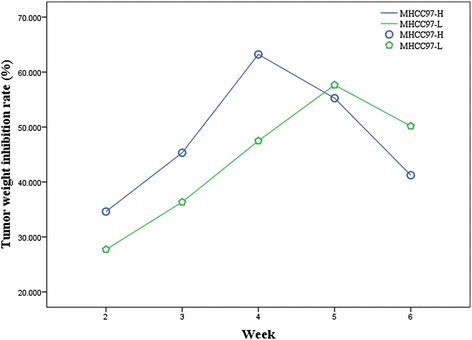
The tumors weight inhibition rate changes of MHCC97-H and MHCC97-L animal model following engraftment of TGFβ-1 transfected hMSC over time. The tumors weight inhibition rate of MHCC97-H animal models (*blue*) was the highest in the fourth week and MHCC97-L (*green*) animal models were the highest in the fifth week by TGFβ-1 transfected hMSC engraftment

Secondly, effects of TGFβ-1 infected hMSC on transplanted tumors in nude mice produced by inoculation of MHCC97-H and MHCC97-L cells were investigated. The experiment lasted for 6 weeks. According to the tumor tissue specimens’ weight in vitro before and after TGFβ-1 infectedhMSC intervention, the tumor weight inhibition rate was calculated (Fig. 9).

Compared with the hMSC without TGFβ-1 gene transfection, the highest tumor inhibition rate of MHCC97-H animal models was observed in the fourth week after TGFβ-1 infected hMSC engraftment, and the tumor inhibition rate gradually reduced with the prolongation of time. The highest tumor inhibition rate of MHCC97-L animal models was observed in the fifth week after TGFβ-1 infected hMSC engraftment, and the tumor inhibition rate gradually reduced with the prolongation of time.

### Relative quantification using real-time PCR analysis

MHCC97-H and MHCC97-L cells served as control groups, and osteopontin (OPN), TGFβ-1, and programmed cell death protein 4 (PDCD4) gene expression levels in hepatoma cells was set as 100 %. So, OPN, TGFβ-1, and PDCD4 gene relative expression levels in hepatoma cells with hMSC and TGFβ-1 gene infected hMSC co-culture groups were shown in the Table 1.

OPN gene relative quantitative expression of MHCC97-H cell were significantly down-regulation after co-cultured with hMSC (*p* < 0.05) and TGFβ-1 gene infected hMSC groups (*p* < 0.01). But OPN gene relative quantitative expression of MHCC97-L cell was only significantly down-regulated after co-cultured with TGFβ-1 gene infected hMSC groups (*p* < 0.01). Transforming growth factor-β1 (TGFβ-1) gene relative quantitative expression of MHCC97-H cell was significantly up-regulated after co-cultured with hMSC (*p* < 0.05) and TGFβ-1 gene infected hMSC groups (*p* < 0.01). TGFβ-1 gene relative quantitative expression of MHCC97-L cell was also significantly up-regulated after co-cultured with TGFβ-1 gene infected hMSC groups (*p* < 0.05). PDCD4 gene relative quantitative expression of MHCC97-H cell was significantly down-regulated after co-cultured with hMSC but was significantly up-regulated after co-cultured TGFβ-1 gene infected hMSC groups (*p* < 0.05). PDCD4 gene relative quantitative expression of MHCC97-L cell was down-regulated after co-cultured with hMSC and up-regulated after co-cultured with TGFβ-1 gene infected hMSC groups. But there were no statistical differences among groups.

## Discussion

Hepatocellular cancer (HCC) remains a common cause of cancer and death worldwide, especially in China. HCC mortality is closely associated with primary organ failure, metastasis, and recurrence after surgical resection. A hot new topic in medical treatment is the use of mesenchymal stem cells (MSC) in treatment of HCC in recent years [7]. Human bone marrow stem cells mainly contain two cell types, including hematopoietic stem cells (HSCs) and bone marrow mesenchymal stem cells (hMSCs). The hMSCs play a multiple role in tumor growth: (1) inhibition of tumor growth, such as in lung cancer and liver cancer; (2) promotion of tumor growth, such as in multiple myeloma and breast cancer; 3) no obvious effect on tumor growth, such as in colon cancer. In 1999, Petersen et al*.* for the first time reported that liver oval cells and liver cells in rat can be differentiated from bone marrow cells [4]. Sato et al*.* divided human bone marrow cells into three types, including hMSCs, CD34 cells, and hMSCs/CD34-cells. These three types of cells were respectively transplanted into rat liver that was injured by allyl ethanol, and hMSCs were the main sources of hepatocytes in necrotic zone. Liver-specific markers were observed in these cells, and cell fusion was not observed [5]. Thereafter, in vivo and in vitro experiments demonstrated that hMSCs can differentiate into hepatocytes or hepatocyte-like cells [8].

So, aggregation of cells, both primary hepatoma cells lines and hMSC, replicate the natural environment of tumor stroma and permit an evaluation of the metastatic behavior and treatment effects of tumor [9]. In our study, all hMSC cells used were restrained within the tenth generation, and these cells were identified using surface antigens and inducing differentiation assay before using it. The hMSCs express CD29, CD44, and CD105 but not CD45 and CD14. Furthermore, these cells can differentiate to adipogenic cells, osteoblasts, and chondrocyte. Hence, the hMSCs were in line with the international standard [6]. MHCC97-H and MHCC97-L are liver cancer cell lines with different metastatic potential, constructed by the Liver Cancer Research Institute of the Fudan University (Shanghai, China) [9, 10].

Moreover, experimental evidence in support of uses of hMSC as vehicles of therapeutic genes is discussed. Because of its regenerative capacity and its particular immune properties, the liver is a good model to analyze the potential of MSC-based therapies. Finally, the potential application of hMSC and genetically modified hMSC in HCC is proposed in view of available evidence [7]. So, our study’s purpose is to observe the effects of hMSC and genetically modified hMSC on different metastatic potential hepatoma cells and xenograft models.

It has been reported [11, 12] that transforming growth factor beta (TGFβ) is a multifunctional cytokine family, which mainly plays roles in regulating cell proliferation, differentiation, and embryo development, promoting the formation of extracellular matrix and inhibiting immune response. Among the members of this family, serum TGFβ-1 is a principal isoform in humans, and it is closely associated with the occurrence and development of tumor. TGFβ-1 plays dual roles in inhibiting and promoting tumor growth. At the early stage of tumorogenesis, TGFβ-1 inhibited normal cell growth and tumorogenesis by suppressing G1/S phase transition. However, the tumor would not be sensitive to TGFβ-1 mediated growth inhibition with the development of tumor. Therefore, based on the hMSC is a good carrier for gene, our aim is to observe effects of the exogenous TGFβ-1 by gene modified hMSC experission on HCC in vitro, especially on the proliferation and invasion ability of tumor tissue or tumor cells.

Results showed that hMSC TGFβ-1 gene transduction rate can reach more than 90 %. Reporter gene eGFP imaging showed that vectors carrying target gene sequences have been successfully imported hMSC. There were not obvious difference on cell morphology between hMSC and hMSC infected TGFβ-1 gene.

So, it is to observe the changes of proliferation and metastasis ability, which the high expression TGFβ-1 of hMSC by gene modified technology is on the different metastatic potential hepatoma cells and xenograft models. In this experiment, we successfully infected TGFβ-1 gene to hMSC, which was high expression of TGFβ-1 cytokine.

This study demonstrated that the hepatoma cells proliferation increased after these cells were co-cultured with hMSC and TGFβ-1 gene transfection hMSC, suggesting that hMSC that can promote the hepatoma cells proliferation and exogenous TGFβ-1 (by TGFβ-1 gene infected hMSC high expression) can also promote the hepatoma cells proliferation. Indeed, qPCR results showed that hepatoma cells expression TGFβ-1 in gene infected hMSC and hMSC groups were higher than in control groups. Our results were consistent with the findings of researchers in Fudan University (Shanghai, China) [10]. Fierro et al*.* study indicated that hMSCs have effects on tumor cells through paracrine mechanism. The hMSC promoted tumor cells proliferation by secreting several factors, including basic fibroblast growth factor (bFGF), platelet derived growth factor B (PDGF-BB), transforming β growth factor 1 (TGFβ-1), and vascular endothelial growth factor (VEGF) [13]. Zhang, et al*.* found also that TGFβ-1 in paracancerous liver tissue was positively correlated with tumor size. Higher production of TGFβ-1 in paracancerous liver tissue was always associated with bigger liver tumors [14]. So, TGFβ-1 gene infected hMSC can still further promote cell proliferation than hMSC without gene infection. Further, from the TGFβ-1 and PDCD4 gene expression by qPCR method, test results showed hMSC promoted hepatoma cells proliferation but had no obvious effect on hepatoma cells apoptosis. So, we can draw a conclusion that the hMSC of infected TGFβ-1 gene can more obviously promote the hepatoma cells proliferation and does not affect the hepatoma cells apoptosis. We are speculating it might be the exogenous TGFβ-1 working. However, we still do not know exogenous TGFβ-1 (by TGFβ-1 gene infected hMSC expression) or endogenous TGFβ-1 (by hepatoma cells expression) the differences of the biological mechanism in promoting hepatoma cells proliferation.

The cells migration change represents the ability changes of cells metastasis. Tumor cells migration breaking through is a vital step for tumor metastasis. When MHCC97-H and MHCC97-L cells were used as control groups in the experiment, our data suggested that hMSC inhibited hepatoma cells migration and made metastatic potential decrease, especially TGFβ-1 genetically modified hMSC. But it was reported [15] that the exogenous TGFβ-1 cytokine could promote tumor migration and metastasis. How to explain this contrary results? It is worth mentioning that OPN expression down-regulated after hepatoma cells were co-cultured with hMSC and TGFβ-1 gene infected hMSC and may be used to explain metastatic potential decrease. In the malignant setting, OPN expression by breast, colorectal, and hepatocellular cancer cell lines stimulates primary tumor proliferation, migration, invasion, angiogenesis, and metastasis. OPN combined with tumor cell surface integrins receptor promotes tumor cell adhesion and extracellular matrix degradation [16, 17]. OPN contains RGD (Arg-Gly-Asp) sequence, which plays key roles in tumor invasion and metastasis. Previous studies found that integrin mediated the adhesions between HCC cell-cell interaction, as well as hepatoma cells and extracellular matrix, respectively. Additionally, integrin was involved in extracellular matrix degradation and hepatocellular carcinoma cell motility (thigmotaxis and chemiotaxis) [18]. Another study showed that the effect of hMSC that influences the hepatoma cells was by means of OPN expression. Hepatoma cells grown with hMSCs for 12 h aggregated into cell clusters resembling hepatospheres. However, cluster formation was inhibited in cultures with ablation of extracellular OPN and was also seen in cell surface α_v_β_3_ integrin blockade with RGD peptide. Hepatospheres are organizing structures found in hepatic cell culture [19]. So, in the absence of OPN expression, cancer cells were less viable away from the primary tumor environment and were less capable of metastases. No doubt, further studies will be necessary to clarify the underlying mechanisms and relationship of TGFβ-1, OPN, and other biological factors.

The paper has carried out a preliminary study for the hMSC intervention in xenograft models on the basis of cytological studies [20]. The most important finding obtained from the study is that the effectiveness of the intervention of hMSCs on HCC with high-metastatic potentials changes with time in animal models. The highest inhibition on HCC was observed at third week after hMSC engraftment. The highest tumor inhibition rate of MHCC97-H animal models was observed in the fourth week, and MHCC97-L animal models was observed in the fifth week after TGFβ-1 infectedhMSC engraftment. It was the same that the tumor inhibition rate gradually reduced with the prolongation of time. hMSC and TGFβ-1 infected hMSC exhibit anti-tumor activity in a time-dependent manner, and the activity against HCC gradually reduces with the prolongation of time.

With regard to the intervention mechanism of hMSC on HCC, studies have reported that hMSC are found to colonize the liver of mouse models of orthotopic liver transplantation and can differentiate into hepatocytes that express albumin; in addition, the hMSCs are mainly found in the marginal area of the tumor and rarely present in tumors or normal liver tissues, and a large necrotic area is observed in tumor tissues following hMSC transplantation [21]. In addition to homogeneous gene characteristics and multilineage differentiation potential, hMSC also have a high efficacy of immunosuppression and anti-infection activity, and the characteristics of migration to inflammatory tissues and remodeling tissues (homing characteristic), which accelerate the repair of injured liver tissues, inhibit immune responses and confer anti-hepatic fibrosis functions [22]. However, prior to this work, to the best of our knowledge, there have been no reports on hMSC anti-tumor activity in a time-dependent manner.

## Conclusions

Our studies demonstrated that hMSC and TGFβ-1 gene infected hMSC can promote the different metastatic potential hepatoma cells proliferation and inhibit different metastatic potential hepatoma cells migration. In the animal models test, we demonstrated that hMSC and TGFβ-1 gene infected hMSC exhibit anti-tumor activity in a time-dependent manner, and the activity against HCC gradually reduces with the prolongation of time. TGFβ-1 cytokine may be mainly factor in HCC proliferation. OPN makes a significant contribution to the changes of hepatoma cells migration. Whether gene modified hMSC as a novel method to intervene the different metastatic potential HCC is the focus of future work.

## Abbreviations

hMSC: Human bone marrow-derived mesenchymal stem cell
HCC: Hepatocellular carcinoma
Real-time PCR: Real-time polymerase chain reaction
OPN: Osteopontin
MHCC97-H: High-metastatic hepatocellular carcinoma cell 97
MHCC97-L: Low-metastatic hepatocellular carcinoma cell 97
SPF: Specific-pathogen-free
TGFβ-1: Transforming growth factor-β1
CD: Cluster of differentiation
PDCD4: Programmed cell death protein 4
